# Mapping the knowledge structure and trends of epilepsy genetics over the past decade: A co-word analysis based on medical subject headings terms

**DOI:** 10.1097/MD.0000000000016782

**Published:** 2019-08-09

**Authors:** Jing Gan, Qianyun Cai, Peter Galer, Dan Ma, Xiaolu Chen, Jichong Huang, Shan Bao, Rong Luo

**Affiliations:** a Department of Pediatrics, West China Second University Hospital, Sichuan University, Chengdu; b Key Laboratory of Birth Defects and Related Diseases of Women and Children (Sichuan University) Ministry of Education, China; c Department of Biomedical and Health Informatics, The Children's Hospital of Philadelphia, PA.

**Keywords:** bibliometric analysis, co-word analysis, genetic epilepsy, mutation, social network analysis

## Abstract

**Introduction:**

Over the past 10 years, epilepsy genetics has made dramatic progress. This study aimed to analyze the knowledge structure and the advancement of epilepsy genetics over the past decade based on co-word analysis of medical subject headings (MeSH) terms.

**Methods:**

Scientific publications focusing on epilepsy genetics from the PubMed database (January 2009–December 2018) were retrieved. Bibliometric information was analyzed quantitatively using Bibliographic Item Co-Occurrence Matrix Builder (BICOMB) software. A knowledge social network analysis and publication trend based on the high-frequency MeSH terms was built using VOSviewer.

**Results:**

According to the search strategy, a total of 5185 papers were included. Among all the extracted MeSH terms, 86 high-frequency MeSH terms were identified. Hot spots were clustered into 5 categories including: “ion channel diseases,” “beyond ion channel diseases,” “experimental research & epigenetics,” “single nucleotide polymorphism & pharmacogenetics,” and “genetic techniques”. “Epilepsy,” “mutation,” and “seizures,” were located at the center of the knowledge network. “Ion channel diseases” are typically in the most prominent position of epilepsy genetics research. “Beyond ion channel diseases” and “genetic techniques,” however, have gradually grown into research cores and trends, such as “intellectual disability,” “infantile spasms,” “phenotype,” “exome,” “ deoxyribonucleic acid (DNA) copy number variations,” and “application of next-generation sequencing.” While ion channel genes such as *“SCN1A,” “KCNQ2,” “SCN2A,” “SCN8A”* accounted for nearly half of epilepsy genes in MeSH terms, a number of additional beyond ion channel genes like *“CDKL5,” “STXBP1,” “PCDH19,” “PRRT2,” “LGI1,” “ALDH7A1,” “MECP2,” “EPM2A,” “ARX,” “SLC2A1,”* and more were becoming increasingly popular. In contrast, gene therapies, treatment outcome, and genotype-phenotype correlations were still in their early stages of research.

**Conclusion:**

This co-word analysis provides an overview of epilepsy genetics research over the past decade. The 5 research categories display publication hot spots and trends in epilepsy genetics research which could consequently supply some direction for geneticists and epileptologists when launching new projects.

## 1 Introduction

Epilepsy is a chronic disease originating from the brain which affects about 0.8% individuals of all ages, especially infants and children, worldwide making it one of the most common neurological diseases globally.^[1]^ Nearly half of the patients diagnosed have an unknown underlying etiology, 30% of which are estimated to be caused by a genetic defect.^[2]^ This rate may be higher in children with early-onset epilepsy.^[3]^ The development of epilepsy genetics can be roughly divided into 3 phases: the channelopathy era, transitional era, and next-generation sequencing era.^[4]^ With the rapid development and broad application of massive parallel sequencing, there has been a dramatic increase in the knowledge of epilepsy genetics during the past decade in terms of etiology, physiopathology, diagnosis, and treatment. While there have been a few reviews and meta-analyses concerning genetic epilepsy, several crucial questions have yet to be interpreted quantitatively and visually in a single article. What are the core themes in existing studies on genetic epilepsy? What are the developing trends in present research? What are the areas of focus in each sub-direction of genetic epilepsy such as etiology, physiopathology, diagnosis, and therapy? To answer these questions, a quantitative and visual analysis is required which reviews and meta-analyses generally cannot accomplish.

Bibliometrics has been broadly applied in the field of quantitative statistical analysis of academic literature to depict the hot spots, trends, and contributions of scholars and journals, as well as countries, which in turn can help scientists keep up to date with a specific scientific field.^[5]^ This analytical technique is playing an increasingly important role in the creation of guidelines and evaluation of research trends.^[6]^ About 40 years ago, Michel Callon invented one of the most important bibliometric methods: the co-word analysis. The co-word analysis can reveal the hot spot of a given field through the use of bundles of professional words or phrases.^[7]^ The basic principle of this technique is based on the co-occurrence frequency of the professional words or phrases of interest in the same article. This frequency can then be implemented to perform hierarchical clustering analysis and to then analyze the relevance of these professional words or phrases.^[8,9]^ The higher co-occurrence frequency of 2 words or phrases in the literature, the closer relationship between the 2 themes.^[10]^ In recent years, this technique has begun to be applied in biological fields, attracting the interest of many medical researchers.^[11,12]^ Medical subjects headings (MeSH) terms present the themes of articles as a set of normalized words. These MeSH terms can then be used to ensure that articles are uniformly indexed by subject. Thus, a co-word network can be mapped by analyzing the co-occurrence frequency of 2 MeSH terms in multiple publications to investigate specific research areas of interest.

Social Network Analysis, also known as network mapping, is a method to study network centralization by analyzing “circles” and “links.” Circles represent MeSH terms and links represent the co-occurrence of these MeSH terms or circles. The size and location of circles depend on the total occurrence frequency of the MeSH terms. The thickness of the links between 2 circles indicates the co-occurrence frequency of MeSH term pairs.^[13–16]^

In this study, a co-word analysis based on MeSH terms was applied to visually map research knowledge structure and trends of epilepsy genetics over the past decade. We hope this analysis may offer some hints for geneticists and epileptologists when launching future projects.

## 2 Materials and methods

### 2.1 Publication search

Literature data were searched and downloaded from PubMed, a free search engine biomedical literature database developed by the US National Center for Biotechnology Information. Most information was obtained from the MEDLINE database on life sciences and biomedical topics.^[10]^ There were no language limitations through the course of our data gathering and analysis. Retrieved studies had to fulfill the following criteria:

(1) the article contained MeSH terms that were related to epilepsy (e.g. “epilepsy” and “seizures”), genetics (e.g. “genetics,” “genes,” “genome,” and “heredity”);
(2) the publication scope was between January 2009 and December 2018; and
(3) only articles, case reports, and reviews were included.

Publications were not included if they met 1 or more of the following exclusion criteria:

(1) the publication was not an article, a case report, or a review, such as news items, book chapters, corrections, and meeting abstracts;
(2) it was a duplication of previous studies;
(3) the publication was not relevant to genetic epilepsy which means literature which did not include both genetics and epilepsy was excluded.

Only epilepsy or seizures papers which discussed “genetics,” “genes,” “genome,” “heredity,” ”copy number variants,” “chromosome,” “ion channel,” “alleles,“ “microRNA,” “long non-coding ribonucleic acids (RNAs),” and “epigenetics” was included. Two investigators independently reviewed all potential studies based on the title, abstract, and, in some cases, the full text. The concordance rate between the 2 investigators was 0.95, which represented a strong agreement.^[17]^ After discussion, a consensus was reached on all items, and this rate rose to 1.

### 2.2 High-frequency MeSH terms extraction

The frequency of occurrence of all MeSH terms extracted from enrolled publications was calculated by VOSviewer software.^[18]^ The Donohue formula^[19]^ was applied to calculate the threshold of high-frequency MeSH terms (T value) as follows: 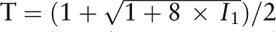, within which *I*_1_ stands for the number of MeSH terms that occurred only once. Consequently, MeSH terms with an occurrence frequency greater than or equal to the T value were defined as high-frequency MeSH terms.

### 2.3 Data analysis and network mapping

Bibliometric information of each publication, such as author, country, language, and publication year were extracted and analyzed via Bibliographic Item Co-Occurrence Matrix Builder (BICOMB) software. Excel and ArcGIS 10.6 software were used to create the graphs. After inputting the data pool into VOSviewer software, the social network analysis of authors and high-frequency MeSH terms and the distribution of high-frequency MeSH terms according to their average appearance were displayed by VOSviewer software with 2-dimensional maps. VOSviewer automatically subgrouped closely related MeSH terms utilizing a default clustering algorithm.^[20]^ The data pool was split by publication year into 2009 to 2013 and 2014 to 2018 groups. To find the dynamic changes in hot spots of genetic epilepsy, the unique high-frequency MeSH terms between the 2 groups were compared through the use of the VOSviewer software's density graph. Finally, the top 20 genes within the MeSH terms pool were highlighted which can provide some indication on all of the current hot spot genes in epilepsy genetics research.

No ethical consent was necessary for this study as it included no experiments with animals or humans.

## 3 Results

### 3.1 Studies inclusion and distribution characteristics

A total of 5422 studies were retrieved from the initial search. After reading the titles, abstracts, and the full-texts in some cases, 6 studies were excluded as duplications, 180 studies were excluded due to publication types (not an article or review), and 51 studies were excluded due to being irrelevant to epilepsy or genetics. A total of 5185 studies were included in the final study, including 4264 articles and 921 reviews.

**Figure 1:**
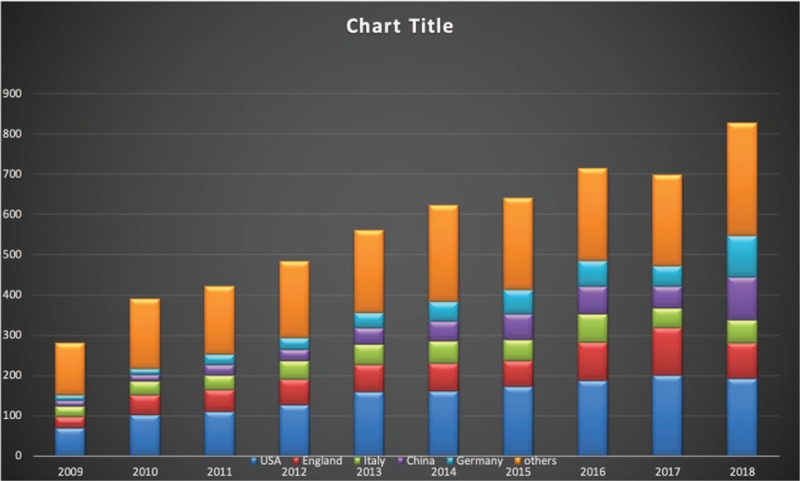
Number of publications on epilepsy genetics from 2009 to 2018. The number of worldwide and the top 5 countries publications on epilepsy genetics research.

**Figure 2:**
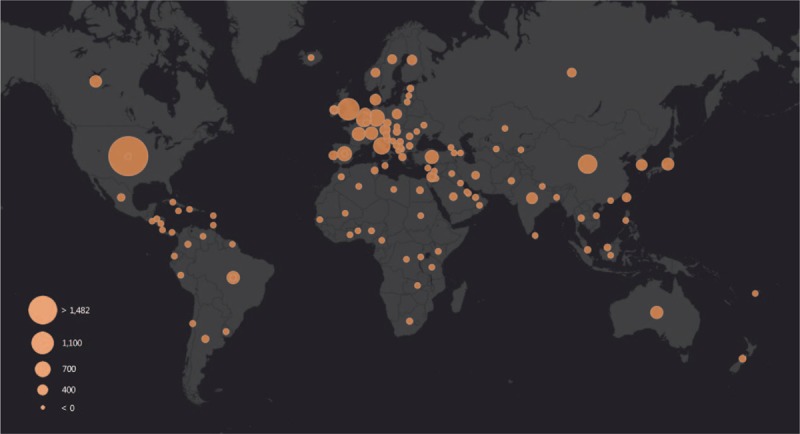
Geographical distribution of publication in epilepsy genetics from 2009 to 2018. The map was created using ArcGIS 10.6 software. The bigger the node is, the higher the productivity this region has. Areas with no circle nodes indicate no data available from these areas.

**Figure 3:**
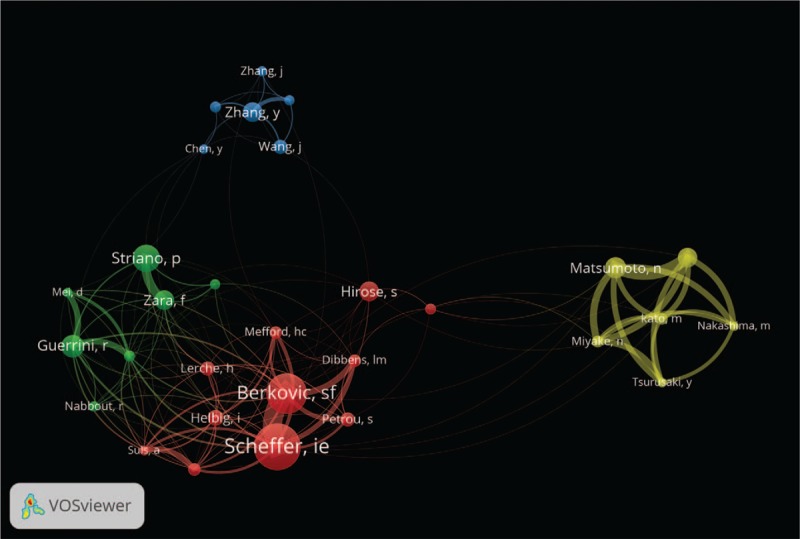
Network visualization map of active authors in epilepsy genetics research. Top 30 authors were visualized. Because of names overlapping, some names might be invisible. The thickness of connecting line between any 2 authors indicates the strength of collaboration. For example, Scheffer, Berkovic, and Helbig existed in one cluster and had the highest percentage of collaboration within this cluster. Petrou, Suls, Ierche, Mefford, and Dibbens were clustered in red since the bulk of their collaboration is with the each other.

Over the past decade, more and more studies have focused on epilepsy genetics research. As shown in Figure 1, the annual publication of articles has gradually increased from 265 in 2009, to 720 in 2018 in the fields of genetic epilepsy. Altogether, 1042 journals have had some involvement in this area of research. The top 10 countries, journals, languages, and authors are displayed in Table 1. Among the top 10 journals, the top 3 are *“Epilepsia*,*” “Epilepsy Research*,*” and “Epilepsy & Behavior*,*”* which contribute more than 13% of the total included publications in this area of research. US authors have contributed to more than one-fourth of epilepsy genetics research studies while authors from US, England, Italy, China, and Germany combined have contributed more than 70% of the research in this field (Figs. 1 and 2). There has been a steady increase in research from England, China, and Germany over the past decade. Journals in English constitute more than 95% of all the publications. There are 4 clusters composed of the top 30 authors (as determined by the total number of publications) in epilepsy genetics depending on the co-authorship network visualization (Fig. 3). The top 10 of these authors were grouped into the red and green clusters, implying a close relationship between each of them.

**Table 1:**
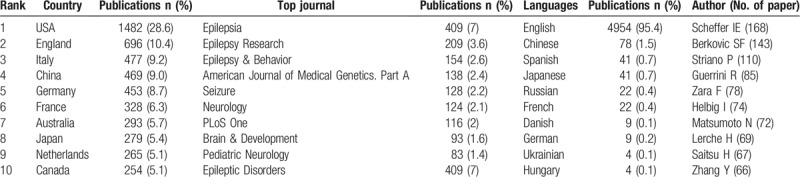
Temporal distribution of publications on epilepsy genetics in PubMed (2009–2018).

### 3.2 MeSH terms with high frequency

In this study, 2628 MeSH terms occurred only once (*I*_1_ = 2628). Using the Donohue formula, the threshold of high-frequency MeSH terms was defined as 73 (T = 73), which meant high-frequency MeSH terms should occur more than 72 times. There were 86 of such MeSH terms in total. A description of the high-frequency MeSH terms is displayed in Table 2. The MeSH terms “epilepsy,” “mutation,” and “seizures” occupied the dominant positions among all the high-frequency MeSH terms, with a frequency of 2303 (8.57%), 1567 (5.83%), and 1126 (4.19%) respectively.

**Table 2:**
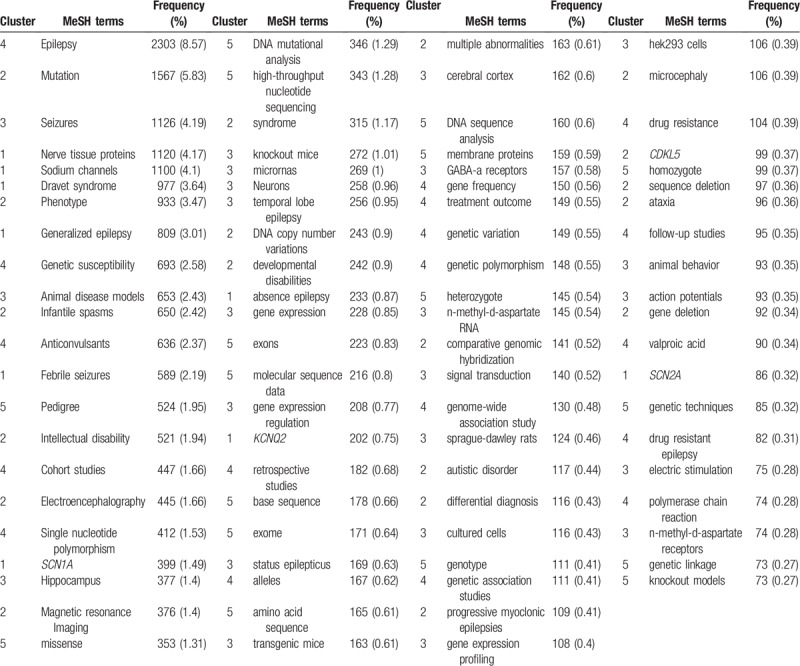
Description of high-frequency MeSH terms in epilepsy genetics field during 2009 to 2018.

### 3.3 Knowledge structure between 2009 and 2018

**Figure 4:**
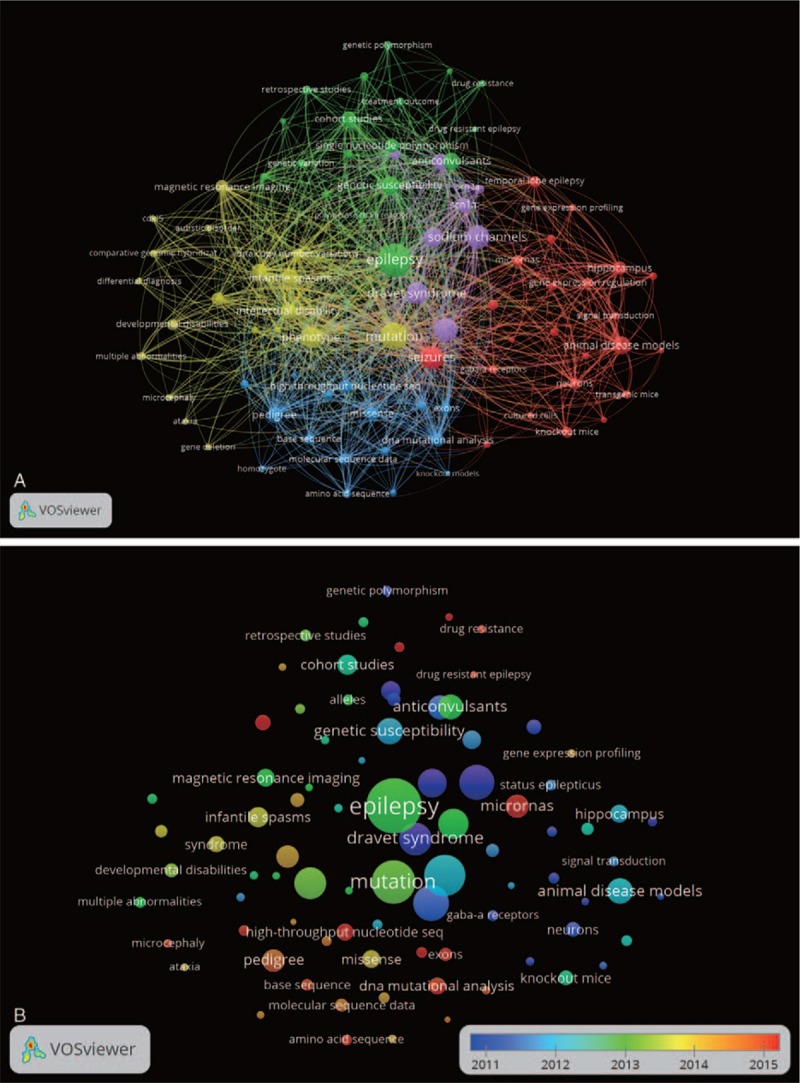
The analysis of MeSH terms. (A). Knowledge network for MeSH terms in epilepsy genetics research, 2009 to 2018. The MeSH terms were subgrouped into 5 clusters according to 5 colors generated by VOSviewer. The nodes represent extracted MeSH terms and the lines stand for relationships between these MeSH terms; the size of the nodes indicates the weight of the MeSH term which is related to the occurrence frequency. The higher the weight of a MeSH term, the larger the node of the item; the thickness of lines reflects the co-occurrence frequency of 2 MeSH terms, thereby representing the links of the 2 MeSH terms. (B). Distribution of MeSH terms based their average appearing time. MeSH terms in blue appeared earlier than those in yellow or red. MeSH = medical subject headings.

Figure 4a displays the MeSH terms network knowledge structure of epilepsy genetics research between 2009 and 2018. The MeSH terms “epilepsy,” “seizures,” and “mutation” are located at the center of this network. All terms were grouped into 5 clusters (Table 3): “ion channel disease” (middle in purple), “beyond ion channel diseases,” “experimental research and epigenetics” (right in red), “single nucleotide polymorphism & pharmacogenetics” (top in green), and “genetic techniques” (bottom in blue). The MeSH terms “sodium channels,” “Dravet syndrome,” “phenotype,” “high-throughput nucleotide sequencing,” “infantile spasms,” and “intellectual disability” are grouped close to the 3 central MeSH terms (“epilepsy,” “seizures,” and “mutation”), and the links of these nodes were centralized.

**Table 3:**
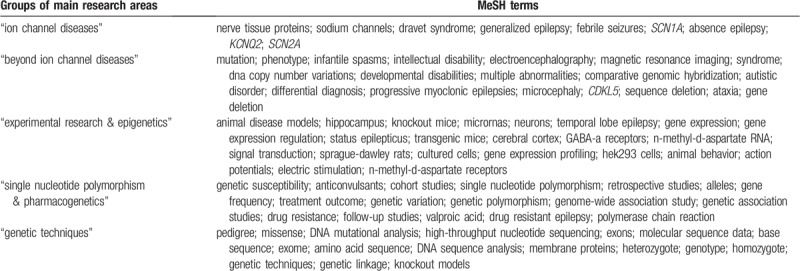
Main research areas in epilepsy genetics during 2009 to 2018.

The distribution of these clusters is displayed in Table 4. The most prevalent research areas were “beyond ion channel diseases” (27%) and “ion channel diseases” (24%). “Experimental research & epigenetics,” “single nucleotide polymorphism & pharmacogenetics,” and “genetic techniques” accounted for 18%, 16%, and 15% of occurrences of MeSH terms, respectively.

**Table 4:**
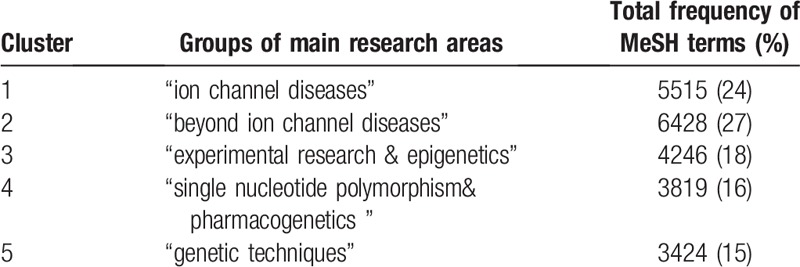
Research group distribution in epilepsy genetics field during 2009–2018.

MeSH terms were colored by VOSviewer according to their average number of appearance (AAY) between 2009 and 2018 across the 5185 related publications (Fig. 4b). Blue colored MeSH terms appeared early, and red-colored MeSH terms appeared later. Before 2012, in the early stage of epilepsy genetics research during the past decade, the most popular topic of research was of “sodium channels” and “Dravet syndrome” (cluster 1, with AAYs of 2011.5 and 2011.7 respectively); “*SCN1A,”* the most common genetic etiology associated with Dravet, had an AAY of 2013.5 with an occurrence of 399. The “beyond ion channel diseases” group's (cluster 2) most common MeSH terms about 6 years ago were “progressive myoclonic epilepsies” (AAY: 2013.0), “*CDKL5”* (AAY: 2013.3), and “sequence deletion” (AAY: 2013.3). More recently in 2014, this cluster's most common MeSH terms were “intellectual disability,” “ deoxyribonucleic acid (DNA) copy number variations,” and “ataxia.” In contrast, the “experimental research & epigenetics” group (cluster 3) displayed a different evolution. In 2012 this group's most prominent MeSH term hot spots were “cultured cells” (AAY: 2012.8) and “Sprague-Dawley rats” (AAY: 2012.6). Recent trends suggest the newest MeSH terms in cluster 3 were “microRNAs” (AAY: 2015.8), “gene expression profiling” (AAY: 2014.3), and “hek293 cells” (AAY: 2014.2). In the “single nucleotide polymorphism & pharmacogenetics” group (cluster 4), the early most prominent MeSH terms were “single nucleotide polymorphism” (AAY: 2011.4) and “genetic susceptibility” (AAY: 2013.2), while the more recent terms were “drug-resistant epilepsy” (AAY:2015.7), “genome-wide association study” (AAY: 2015.4), and “treatment outcome” (AAY: 2014.9). Within the “genetic techniques” group (cluster 5), “homozygote” (AAY: 2013.8), “heterozygote” (AAY: 2014.0), and “molecular sequence data” (AAY: 2014.1) were the early hot spot terms. Later trends suggest the terms “high-throughput nucleotide sequencing” (AAY: 2015.8), “exome” (AAY: 2015.4), and “exons” (AAY: 2105.2).

### 3.4 Unique high-frequency MeSH terms in 2009 to 2013 and 2014 to 2018

**Figure 5:**
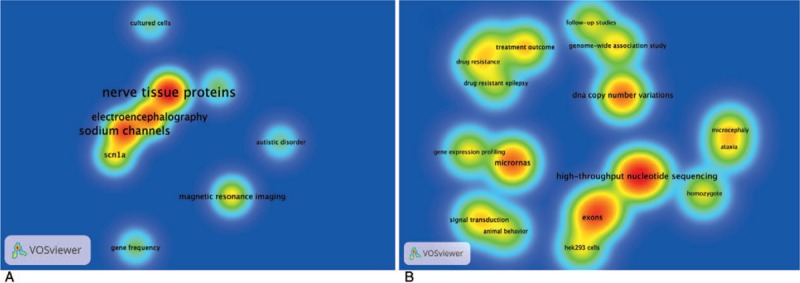
Density visualizations of VOSviewer maps. 2009 to 2013 group (A), 2014 to 2018 group (B). After splitting the data pool into 2009 to 2013 group and 2014–2018 group, we drew the density visualizations of the 2 groups independently by the unique high-frequency MeSH terms according to the T value respectively.

Figure 5 displays density visualizations of unique high-frequency MeSH terms in 2009 to 2013 (A) and 2014 to 2018 (B) respectively which can give us some hints to the dynamic changes in the hot spots of genetic epilepsy research. In Figure 5A, “electroencephalography,” “nerve tissue proteins,” “magnetic resonance imaging,” “sodium channels,” and “*SCN1A*” were the prevalent MeSH terms. In Figure 5B, “high-throughput nucleotide sequencing microRNAs,” “exons,” “exome,” “DNA copy number variations,” “drug-resistant epilepsy,” “treatment outcome,” and “gene expression profiling” emerged which indicates that “genetic techniques” and “experimental research & epigenetics” were becoming integral to genetic epilepsy.

### 3.5 Top 20 genes in MeSH terms pool

**Figure 6:**
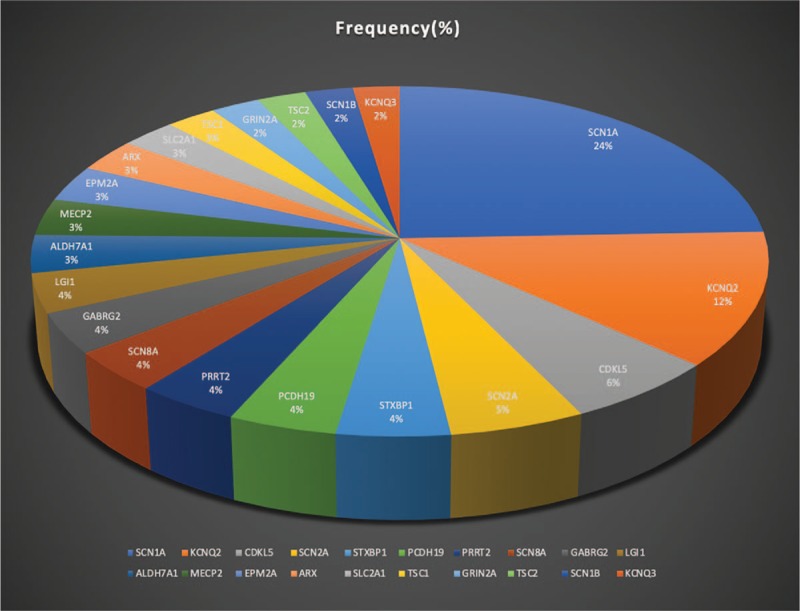
Frequency composition ratio of top 20 genes in MeSH terms pool of epilepsy genetics field during 2009 to 2018. MeSH = medical subject headings.

Only *“SCN1A,” “KCNQ2,” “CDKL5,” and “SCN2A”* appeared in the high-frequency MeSH terms. This, however, could not represent all the hot spot genes in epilepsy genetics research. Consequently, a graph was composed to depict the component ratio of the top 20 genes in the MeSH terms pool of epilepsy genetics field during 2009–2018 (Fig. 6). Ion channels such as *“SCN1A,” “KCNQ2,” “SCN2A,” “SCN8A”* accounted for nearly half of all occurrences. Beyond ion channel genes such as *“CDKL5,” “STXBP1,” “PCDH19,” “PRRT2,” and “LGI1”* were the most common terms.

## 4 Discussion

Generally, there was a steady growth of publications related to epilepsy genetics research from 2009 to 2018 around the world. The United States ranked as the highest contributor to epilepsy genetics research. Moreover, the number of the publications from American, English, Italian, Chinese, and German authors accounted for more than 70% of the total publications, suggesting that the scientists from these 5 countries took leading position in epilepsy genetics research likely in conjunction with some of the most powerful research institutions in the world in their respective countries. Of note when considering the authors, Scheffer and Berkovic from Australia and Striano and Guerrini from Italy together have published the most papers on epilepsy genetics. These scientists are leaders in their field, and their publications play an important role in epilepsy genetics research in the present and future which, in turn, can aid other researchers in the design of novel experiments. With respect to journals, *Epilepsia* has had by far the most publications in this area of research (409 publications in the past decade). In addition, *“Journal of Epilepsy Research,” “Epilepsy & Behavior,” “American Journal of Medical Genetics Part A,” and “Seizure”* were the other primary journals publishing epilepsy genetics papers. Most of the journals (95.4%) are published in English.

The “ion channel diseases” cluster (including the terms “nerve tissue proteins,” *“SCN1A,” “KCNQ2,”* “febrile seizures,” etc) is one of the most important groups in genetic epilepsy territory. Ion channel diseases appeared in the early stages of epilepsy genetics research and have continued to flourish for the past 10 years. It has been estimated that nearly 25% of genes identified in the epilepsy genetics belonged to ion channels.^[21]^ Though the total frequency of MeSH terms in this cluster was a little less than cluster 2 (”beyond ion channel diseases“) due to the numbers of MeSH terms total in the former group. The average occurrences of MeSH terms in “ion channel diseases” were the highest among the 5 groups. This suggests “ion channel diseases” still occupies a leading position in epilepsy genetics research.

“Sodium channels” has a very strong and close link with the MeSH terms “epilepsy,” “seizures,” “mutation,” and “Dravet syndrome.” Sodium channels have always been at the core of ion channel disease research over the past decade. This is due not only to the fact that sodium channels were the first discovered epileptic genes, but also because they make up the highest constituent ratio in epilepsy genetics.^[22]^ Other channel diseases also have attracted increasing attention in recent years such as *“KCNQ2,” “KCNQ3,” “GABRG2,” “GABRA1,” “GRIN2A,” “GRIN2B,” “CHRNA4,” “CHRNB2,” and “CHRNA2”*^[1,21,23–25]^ (Fig. 6) each of which can also result in varying forms of epileptic encephalopathy, syndromes, and seizures. Most of the early discovered epileptic genes were ion channels which consequently gave rise to the misconception that all of epilepsy genetics belonged to a family of channelopathies.^[26,27]^ No doubt, this theory has evolved significantly, nevertheless, ion channel diseases still contribute a major proportion of known genetic epilepsies. As seen in Figure 6, *“SCN1A”* and *“KCNQ2”* accounted for the greatest composition ratio in this study; this has a high concordance rate with Lindy's clinical research.^[1]^

A huge host of projects have been conducted to unraveling the hidden mechanics of the cellular, molecular, and neuronal network of ion channel diseases with the aim to offer precision therapy for genetic epilepsy.^[21,28–31]^ We expect a rise in ion channels research likely greater than most may expect. This, in turn, could shape the architecture of genetic epilepsies more than ever before. Nevertheless, channelopathy studies will likely continue to occupy the core position in epilepsy genetics frameworks. Nowadays, the trend of research on ion channel diseases aims at developing therapeutic strategies based on disease mechanisms through the combination of tools such as genetic models, functional studies, and new molecular technologies.^[21]^

In our co-word analysis, the largest cluster is the “beyond ion channel diseases” group which includes “mutation,” “infantile spasms,” “phenotype,” “intellectual disability,” and “DNA copy number variations” which contribute greatly to the etiology of genetic epilepsy.^[32–34]^ The definition of mutation in the MeSH database can be described as a permanent change in the nucleotide sequence of DNA and which is transmitted to daughter cells and to offspring. Nowadays, genetic counselors in clinical practice have called for the word “mutation” to be replaced by “variant” due to the frequency of confusion in its meaning.^[35]^ In this study, “mutation” was still used to represent all the terms below it in the ontology of the MeSH database, consisting of: “missense mutation,” “chromosome aberrations,” “sequence deletion,” “gene duplication,” “point mutation,” “frameshift mutation,” and more.

“Beyond ion channel diseases” have become one of the main focuses of research in epilepsy genetics over the past 10 years. In fact, it has long been recognized that “beyond ion channel diseases” are associated closely with epilepsy genetics which can be seen in the network map (Fig. 4). The research on “beyond ion channel diseases” ranges from “phenotypes” to “infantile spasms,” “intellectual disability,” “DNA copy number variations,” “developmental disabilities,” and *“CDKL5”* which seems continually to be the focus in the future. Additionally, according to Figures 4B and 5B, studies concerning “DNA copy number variations,” “multiple abnormalities,” “ataxia,” and “microcephaly” have risen. These findings suggest that research on the phenotypes may trend towards “Beyond ion channel diseases.” “Gene deletion” and “chromosome deletion” also appeared in this network. This demonstrates that genetic variants are being reported in growing numbers in recent years, likely benefiting from the increasing use of advanced genetic techniques in the clinic.

Cluster 1 (“ion channel diseases”) and cluster 2 (“beyond ion channel diseases”) exhibited some overlap. For example, “infantile spasms,” “phenotype,” “intellectual disability,” “DNA copy number variations,” and “gene deletion” also have a relationship with “ion channel diseases” (cluster 1). Cluster 2 had a strong link with “infantile spasms” and “intellectual disability” resulting from non-ion channel abnormalities such as *“CDKL5,”* “gene defect,” and “copy number variations”. Undoubtedly, ion channels have enabled us to uncover many epileptogenesis.^[36]^ However, according to the statistics and network visualization map of this study, more recent studies have shifted to the field beyond the ion channels, and, with the popularization of new genetic techniques, we are able to trace physiopathology of more complex genetic epilepsies, regardless of their rarity,^[37]^ which makes epilepsy gene discovery one of the most fascinating and productive fields of human genetics in the near future. For example, in this study *“CDKL5,” “STXBP1,” “PCDH19,” “PRRT2,” “LGI1,” “ALDH7A1,” “MECP2,” “EPM2A,” “ARX,”* and *“SLC2A1”* were among the most popular terms associated with “beyond ion channels” (Fig. 6).

“Animal disease models,” “knockout mice,” “temporal lobe epilepsy,” “hippocampus,” “gene expression regulation,” and “transgenic mice” are the primary MeSH terms that are associated with the cluster “experimental research & epigenetics.” This network suggests that both experimental research and epigenetics play important roles in epilepsy genetics research. In order to unravel the complicated genetic structure of epilepsy, more and more in vivo and in vitro models are being developed.^[38,39]^ This is reflected by the MeSH terms “animal disease models,” “neurons,” “transgenic mice,” “cultured cell,” and “hek293 cells” in cluster 3. These are promising techniques to validate the causation of new genes and to discover powerful and targeted therapeutic schemes for genetic epilepsy. For example, standard knockout and knock-in rodent models have proven to be very useful to uncover loss-of-function and gain-of-function mutations.^[40–42]^ As an “on top of” or “in addition to” the traditional genetic basis for inheritance, epigenetics plays a vital role in the development of epilepsy through a mechanism of gene-expression regulation in forms of DNA methylation, post-translational histone modification, and non-coding RNA.^[43]^ In this study, MeSH terms related to epigenetics contained “gene expression regulation,” “gene expression,” and “microRNAs” which were predominantly studied in the hippocampus of temporal lobe epilepsy with status epilepticus. Given that epigenetic regulation determines gene expression or silencing with time and space specificity, epigenetic studies may facilitate potential novel therapeutic strategies for genetic epilepsy.^[44,45]^ “MicroRNAs” have become increasingly popular hot spots demonstrated by the trend in cluster 3 (Figs. 4B and 5B). Novelties like “long non-coding RNA,” “competitive endogenous RNA,” and “circular RNA,” albeit not appearing in this study, have become very popular as of late implying possible new trends in epigenetics in the coming years.^[46,47]^

Cluster 4 in our network was defined as “single nucleotide polymorphism & pharmacogenetics” whose core MeSH terms were: “genetic susceptibility,” “anticonvulsants,” “cohort studies,” “single nucleotide polymorphism,” “genetic association studies,” “treatment outcome,” “follow-up studies,” and “drug-resistant epilepsy” with new, emerging trends in “genome-wide association study,” “treatment outcome,” and “valproic acid” (Fig. 4B). “Genome-wide association studies” could offer an unbiased, non-candidate-driven approach for risk factors of epilepsy genetics on a genome-wide basis, typically focusing on associations between single nucleotide polymorphism and identifying robust common variants with small hereditary contribution,^[34]^ such as “*CHRM3,*” “*VRK2*,” “*ZEB2*,” “*PNPO*,” “*PCDH7*,” “*SCN1A*,” “*GABRG2*,” “*ATP1A3*,” and “*KCNQ2*”.^[34,48,49]^ Moreover, single nucleotide polymorphism plays a vital role in pharmacogenetics with important implications for clinical practice,^[50–52]^ which may affect drug response, drug pharmacokinetics, drug pharmacodynamics, adverse drug reactions, and precision medicine treatment. For example, *HLAB∗1502* and *HLA-A∗3101* variants were the milestones in pharmacogenetics of epilepsy.^[53]^ As seen in Figure 4B, research on anticonvulsants like “valproic acid” and “treatment outcome” of epilepsy genetics was the recent hot spot of cluster 4, albeit precision medicine treatment is still in its infancy. Although there are many advances in pharmacogenetics of epilepsy with robust evidence, much more findings still need to be validated further. This requires large cohorts, functional study, as well as international and interdisciplinary collaboration.

In this study, the “genetic techniques” group was the cluster with the strongest potential in the future as seen in Figures 4B and 5B. Its core MeSH terms were all related to genetic techniques such as “pedigree,” “missense mutation,” “high-throughput nucleotide sequencing,” “molecular sequence data,” “exome,” “exons,” and “DNA mutational analysis.” Although the total occurrence frequency of these MeSH terms is not as high as those related to “ion channel” or “beyond ion channels diseases,” these MeSH terms will gradually become core research themes in epilepsy genetics research. Moreover, both “high-throughput nucleotide sequencing” and “exons” were found to have been extremely prevalent in epilepsy genetics research throughout recent years (Fig. 5B). Undoubtedly, the most prominent recent advance in the human genetics in the past decade benefited from the development and world-wide application of genetic techniques such as array-comparative genomic hybridization, genome-wide association study, and high-throughput nucleotide sequencing, including whole genome sequencing and whole exome sequencing.^[54]^ Through the combination of new gene-screening techniques, international alliances, and powerful bioinformatics tools, we have entered a massive parallel sequencing era which has led to an index increase in the number of genes identified in epilepsy genetics during the past decade.^[34,55]^ This, in turn, generates a huge amount of new data needing comprehensive and insightful interpretation for clinical dimensions. MeSH terms like “genotype,” “phenotype,” “pedigree,” “missense,” and “heterozygote” were useful for this purpose (also hot spots in this network, 4a). “Genotype-phenotype correlations” were gradually used for depicting the statistical relationship between the genetic composition of an individual and characteristics of interest on the basis of computational methods.^[56,57]^ Although “Genotype-phenotype correlations” was absent from the list of high-frequency MeSH terms, it is the new trend in the future. With the prosperity of big data from genetic techniques, phenotyping has become the bottleneck of collaborations among clinicians, medical informaticists, and statistical geneticists. Nowadays, more and more scientists are working on developing strategies to overcome this, integrating genomic data with epilepsy-specific Human Phenotype Ontology (HPO) in an attempt to better understand how genes and phenotypes are linked, which hopefully will lead to novel therapeutics.^[58–60]^

There were several limitations of this study. Firstly, all the papers were not weighted according to their quality, consequently, a few low-quality papers could have the same weight as exceptional high-quality articles. Secondly, more specified MeSH terms were chosen. For example, “infantile spasms,” “Dravet syndrome,” and “Lennox Gastaut Syndrome” were used instead of “epileptic encephalopathy” which should have appeared in this study but was not found in the high-frequency MeSH term pool. Lastly, this study was based on high-frequency MeSH term co-word analysis, which would exclude the new emerging topics with low occurrence. Thus, new emerging topics and multiple databases should be analyzed in future studies.

## 5 Conclusion

In summary, bibliometric information, co-word analysis, and social network analysis were applied to provide a comprehensive examination of research focuses and trends in epilepsy genetics research architecture during 2009 to 2018. Research on epilepsy genetics experienced accelerated development during the past decade. Our work demonstrates the 5 research categories according to publication trends on epilepsy genetics research. Besides ion channel diseases, beyond ion channel diseases and genetic techniques have gradually been growing research cores, so to have the MeSH terms “intellectual disability,” “infantile spasms,” “phenotype,” “exome,” “DNA copy number variations,” and “application of next-generation sequencing.” In the future, gene therapies, treatment outcome, and genotype-phenotype correlations deserve further development, and more studies are required in the bioinformatic analysis of genetic epilepsy. This study may supply some potential direction for geneticists and epileptologists when launching new projects.

## Acknowledgments

Thank you to Mrs Haixia Liu, statistical and bioinformatical experts from central south university of China, for consultation regarding statistics and bibliometrics (e-mail: 803255@csu.edu.cn).

## Author contributions

**Conceptualization:** Jing Gan, Qianyun Cai.

**Data curation:** Jing Gan, Dan Ma, Xiaolu Chen.

**Data curation:** Jing Gan, Dan Ma, Xiaolu Chen.

**Formal analysis:** Jing Gan, Jichong Huang, Shan Bao, Qianyun Cai, Xiaolu Chen.

**Funding acquisition:** Jing Gan, Dan Ma.

**Investigation:** Jing Gan, Qianyun Cai, Dan Ma, Rong Luo.

**Methodology:** Jing Gan, Jichong Huang, Shan Bao, Qianyun Cai, Dan Ma, Xiaolu Chen, Jichong Huang, Shan Bao.

**Project administration:** Jing Gan.

**Resources:** Qianyun Cai, Dan Ma, Xiaolu Chen, Jichong Huang, Shan Bao, Rong Luo.

**Software:** Jing Gan, Qianyun Cai, Peter Galer, Dan Ma, Xiaolu Chen, Jichong Huang.

**Supervision:** Jing Gan, Qianyun Cai, Xiaolu Chen, Shan Bao, Rong Luo.

**Validation:** Jing gan, Jichong Huang.

**Visualization:** Jing gan, Qianyun Cai, Dan Ma, Xiaolu Chen, Jichong Huang.

**Writing – original draft:** Jing gan, Qianyun Cai, Peter Galer, Rong Luo.

**Writing – review & editing:** Qianyun Cai, Peter Galer, Rong Luo.

Jing Gan orcid: 0000-0001-5255-6324.
